# Voltage-gated potassium channels and genetic epilepsy

**DOI:** 10.3389/fneur.2024.1466075

**Published:** 2024-10-07

**Authors:** Yiting Zheng, Jing Chen

**Affiliations:** Department of Neurology, Children’s Hospital of Nanjing Medical University, Nanjing, China

**Keywords:** epilepsy, seizure, potassium channel, potassium channel gain of function, genetic diagnosis

## Abstract

Recent advances in exome and targeted sequencing have significantly improved the aetiological diagnosis of epilepsy, revealing an increasing number of epilepsy-related pathogenic genes. As a result, the diagnosis and treatment of epilepsy have become more accessible and more traceable. Voltage-gated potassium channels (Kv) regulate electrical excitability in neuron systems. Mutate Kv channels have been implicated in epilepsy as demonstrated in case reports and researches using gene-knockout mouse models. Both gain and loss-of-function of Kv channels lead to epilepsy with similar phenotypes through different mechanisms, bringing new challenges to the diagnosis and treatment of epilepsy. Research on genetic epilepsy is progressing rapidly, with several drug candidates targeting mutated genes or channels emerging. This article provides a brief overview of the symptoms and pathogenesis of epilepsy associated with voltage-gated potassium ion channels dysfunction and highlights recent progress in treatments. Here, we reviewed case reports of gene mutations related to epilepsy in recent years and summarized the proportion of Kv genes. Our focus is on the progress in precise treatments for specific voltage-gated potassium channel genes linked to epilepsy, including KCNA1, KCNA2, KCNB1, KCNC1, KCND2, KCNQ2, KCNQ3, KCNH1, and KCNH5.

## Introduction

Recent studies have identified more than 70 genes associated with epileptic phenotypes (1). Genetic factors contribute to approximately half of epilepsy cases, so understanding the pathogenic effect of genes and considering them during the diagnosis and treatment of epilepsy is essential. Potassium channels, which ubiquitously exist in nearly all parts of life, perform diverse but vital functions (2). Mutations in potassium channels have been implicated in several conditions, including various epilepsies, and proved to play a crucial role in epilepsy treatments.

Potassium ion channels facilitate K^+^ to move swiftly and selectively across the cell membrane along an electrochemical gradient (3). Based on the number of transmembrane domains in each subunit and their gating mechanisms, potassium channels are categorized into three structural families: Voltage-activated and Ca2^+^-activated K^+^ channels, the ‘leak’ K^+^ channels, and the ‘inward rectifiers.’ Each of the three groups’ content is further divided into subfamilies (4).

The voltage-gated potassium channel superfamily is the largest and most diverse family of ion channels, comprising approximately 80 genes divided into 12 subfamilies (Kv1 to Kv12) in humans, playing key roles in various aspects of neuronal signaling, including the regulation of electrical excitability (5). Structurally, Kv channels typically consist of tetrameric *α*-subunits, each containing six membrane-spanning α -helices (S1–S6), S1–S4 segments form voltage-sensing domain, while the S5-S6 segments contribute to pore domains of the channel. In voltage-gated channels, heterozygous mutations are implicated in epileptic disorders (5). Mutations primarily affect the main pore-forming subunit(s), as in regulatory ones. These mutant channel proteins are commonly associated with either reduced expression of the channel on the plasma membrane or altered channel kinetics *in vitro* (1).

Recent research has shown that both gain-of-function and loss-of-function mutations of Kv channels can result in epilepsy (6). Previously, increased neuronal excitability in epilepsy was linked to reduced channel activity, caused by decreased open probability, altered voltage-dependent activation, or reduced surface expression. Clinically, channel openers have been used to target to these loss-of-function mutate channels. However, exome sequencing has discovered several cases linked to channel gain-of-function variants. Increased potassium channel activity leads to similar phenotypes, complicating the diagnosis and treatment of epilepsy.

In studies of genetic epilepsy, there are several model organisms offering their unique benefits. Mice have close neurobiological similarities to humans and the ability to undergo genetic modifications, thus they are preferred models in the study of spontaneous and induced seizures (7). Mice models including Kv1.2 (8) and Kv2.1 knock-out mice (9), methylazoxymethanol-exposed rats (10), are helpful in understanding the mechanisms of epilepsy related to voltage-gated potassium channel mutations, they are also used to test potential treatments. Zebrafish are often used in large-scale genetic screens because they have genetic manipulation and are transparent during development. In some research, drosophila is used to study genetic basis of epilepsy (11), *C. elegans* can offer simpler neural models for basic genetic screening though they have limits in mimicking human channel function (12). Pluripotent stem cell (iPSC) technology can also provide a reliable *in vitro* model for ion channel dysfunction (13).

**Figure 1:**
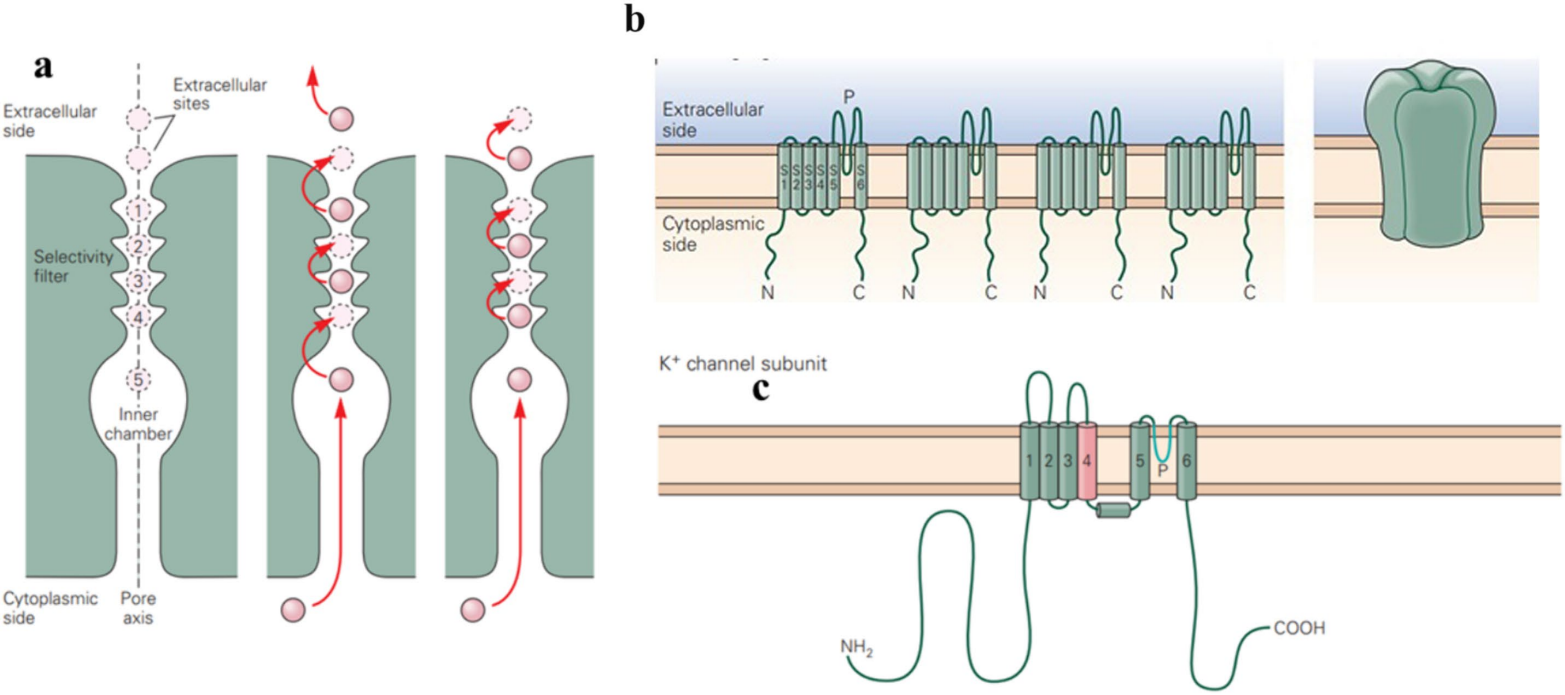
Potassium ion channels are integral membrane proteins that selectively allow potassium ions (K^+^) to pass through the membrane. (a) Ion permeation through the channel involves a pair of K^+^ ions hopping between binding sites in the selectivity filter. The entry of an ion causes ions in the outer configuration (sites 1 and 3) to move outward, expelling an ion and shifting the remaining ions to the inner configuration (sites 2 and 4). The movement of K^+^ across the membrane contributes to various physiological functions, such as repolarizing the membrane after action potentials. (b,c) Voltage-gated potassium channels are composed of four subunits, with six transmembrane segments and a pore-forming P-region. (Adapted from Principles of Neural Science Fifth Edition by Eric R. Kandel and James H. Schwartz and Thomas M. Jessell and Steven A. Siegelbaum and A. J. Hudspeth, McGraw-Hill Companies, New York, USA).

**Figure 2:**
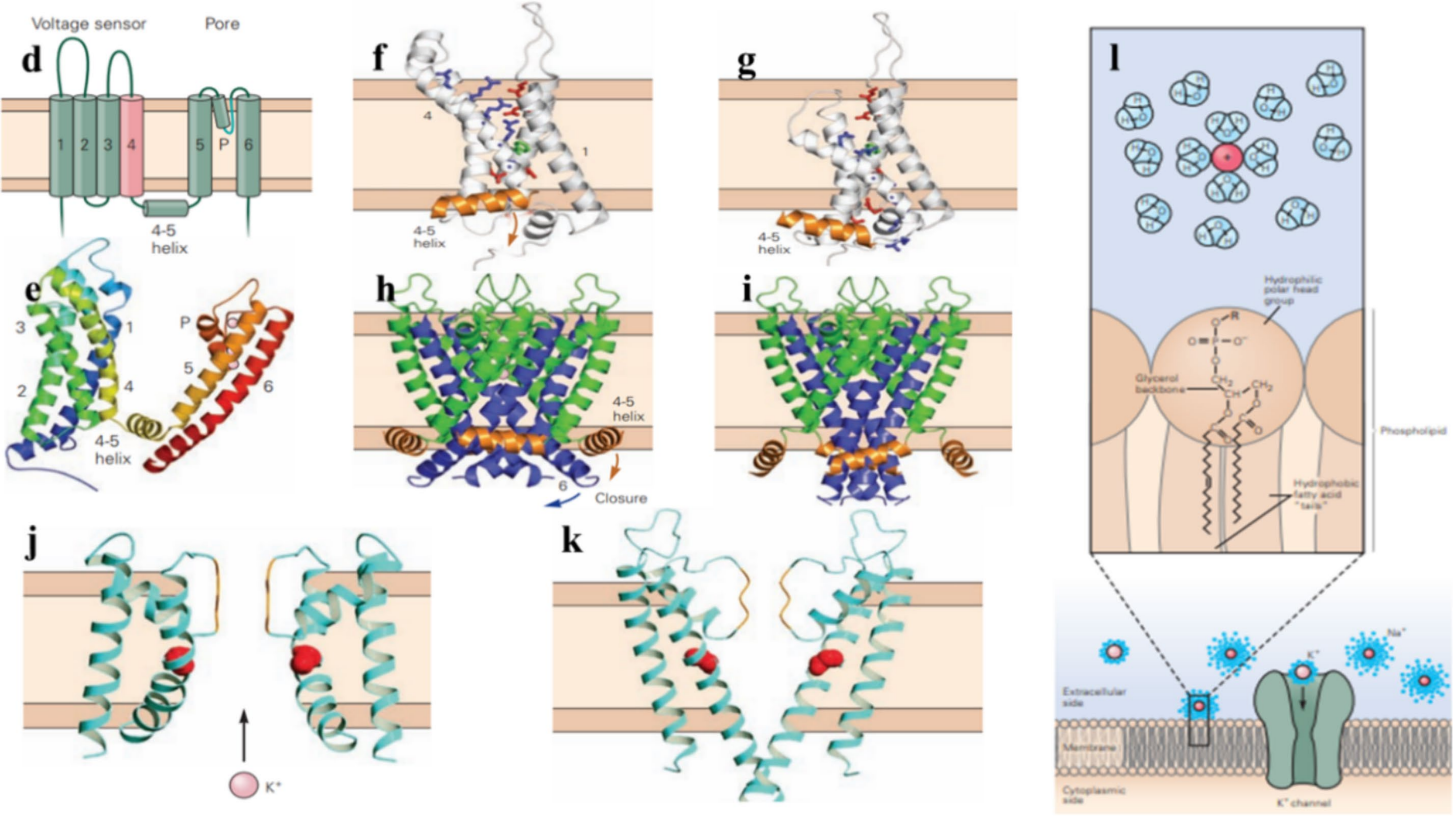
Potassium channels help regulate the electrical excitability of cells, control the duration and frequency of action potentials, and maintain the resting membrane potential. (d) A voltage-gated potassium channel subunit contains six transmembrane *α*-helixes (S1–S6), a short α-helix (the P helix), as well as an α-helix on the cytoplasmic side of the membrane that connects transmembrane helixes S4 and S5 (4–5). (e) Side view of the tetrameric voltage-gated potassium channel. (f,g) Voltage-gated potassium channels open or close in response to changes in the membrane potential, the S1–S4 voltage-sensing domain shifts between the external and internal halves of the membrane, influencing the movement of the S4–S5 coupling helix. (h,i) In voltage-gating, membrane repolarization makes the S4–S5 coupling helix move downward, the S6 inner helix bends at its glycine hinge to close the channel gate. (j,k) In the closed state, the inner helices of the pore are too narrow for K+ to pass through. In the open state, these helices bend and widen the pore to allow ion flow through it. (l) Potassium ion channels are highly selective for K^+^ over other ions, the permeability of them is determined by the interaction of ions with water, the membrane lipid bilayer, and ion channels. (Adapted from Principles of Neural Science Fifth Edition by Eric R. Kandel and James H. Schwartz and Thomas M. Jessell and Steven A. Siegelbaum and A. J. Hudspeth, McGraw-Hill Companies, New York, USA).

This article focuses on summarizing recent clinical developments concerning the critical voltage-gated potassium (KV) family-related epilepsies from the KCNA-, KCNB-, KCNC-, KCND-, KCNQ- and KCNH-subfamilies (Figures 1, 2 and Table 1).

**Table 1:** The genes in the article and related diseases and treatments of them.

Gene	Related disease	Treatment
KCNA1	Epilepsy, Episodic Ataxia Type 1, SUDEP	carbamazepine, 4-aminopyridine, SUDEP prevention strategies
KCNA2	Epilepsy, Ataxia, Neurodevelopmental Disorders	4-Aminopyridine, ketogenic diet, gene therapy
KCNB1	Developmental and Epileptic Encephalopathy (DEE), Autism	Valproic acid, Angiotensin II, neuroprotective therapies
KCNC1	Epileptic Encephalopathy, Myoclonus Epilepsy, Ataxia	Small molecule Kv3 activators, pharmacological treatments
KCND2	Autism, Epilepsy, Cortical Malformations	Antagonizing miR-324-5p, Saikosaponin A
KCNQ2	Benign Familial Neonatal Epilepsy (BFNE), Encephalopathy	Carbamazepine, KCNQ openers like retigabine, cannabidiol, SCR2682
KCNQ3	Benign Familial Neonatal Epilepsy (BFNE), Developmental Delay	Similar treatments to KCNQ2, selective channel openers
KCNH1	Epileptic Encephalopathy, Autism	Benzodiazepine, phenytoin, valproate
KCNH5	Developmental and Epileptic Encephalopathy (DEE), Autism	Valproate, terbutaline

## KCNA1 and KCNA2

The KCNA1 gene encodes the voltage-gated potassium channel Kv1.1, which plays a crucial role in nerve cell repolarization following an action potential, thereby contributing to neuronal excitability, firing rate, and neurotransmitter release (8). Kv1.1 channels are expressed in the central and peripheral nervous systems, prominently in the hippocampus, cerebellum, neocortex, and peripheral nerves (14). Except in the brain, Kv1.1 proteins are also found in cardiac tissues (15). KCNA2 gene encodes Kv1.2, another prominent voltage-gated potassium channel responsible for regulating cellular excitability and action potential propagation (16). Kv1.2 channels are typically localized with Kv1.1 along axons and axon terminals at presynaptic sites (17). KCNA2 mutations lead to dominantly inherited episodic ataxia, mild infantile-onset seizures, and later generalized and focal epilepsies in the setting of average intellect (18). Abnormal function of Kv1.1 and Kv1.2 channels can lead to changes in neuronal excitability, leading to epilepsy and other neurological disorders.

The first K^+^ channel gene directly linked to a human disease was KCNA1 (15). Research showed that mutations in KCNA1 on chromosome 12p13 are pathogenic and result in epilepsy in EA1 (19). KCNA1 mutations may also lead to a sudden, unexpected death in epilepsy (SUDEP). Moore et al. used Kv1.1 null mouse to stimulate SUDEP in patients who experienced ictal and postictal bradycardia. All null mice with a normal rhythm died following seizures (20). This finding highlights the potential for brain-heart channelopathies can increase death risk by brain-driven mechanisms alone without a functionally compromised heart, suggesting seizure control targets to gene mutations have an essential position in SUDEP prevention (21).

Both loss-of-function and gain-of-function mutations in KCNA1 can cause epilepsy. The loss of Kv1.1 from its normal localization leads to increased excitability in the CA3 recurrent axon collateral system, contributing to the epileptic phenotype of limbic components (22). KCNA1 pore loss-of-function mutations in the selectivity filter or the proline residues are proven to cause epileptic phenotypes, the P403A variant in Kv1.1 contributes to smaller potassium currents, positively-shifted voltage-dependent activation and slower activation kinetics compared to the wildtype, leading to drastically reduced channel activity. Meanwhile, the A261T variant causes milder epileptic phenotypes such as mild, carbamazepine-sensitive, childhood-onset focal epilepsy (23) without intellectual disability (8). Channels with the p.A261T variant exhibited a gain-of-function effect on the activation process’s hyperpolarizing shift and the voltage sensitivity increase. It is believed that gain-of-function variants causing neuronal hyperexcitability reduce not only excitability but also neurotransmitter release via a modulatory effect on the presynaptic action potential waveform (24), faster action potential repolarization may lead to sodium channels repriming, thus increasing firing frequency and synchronization, cases of mild, drug-responsive focal epilepsy have been observed (23).

KCNA2 mutations are implicated in human neurodevelopmental disorders through two different mechanisms: predicting either hyperexcitability or electrical silencing of KV1.2-expressing neurons. A similar loss of function effect as KCNA1 for the corresponding substitution P405L in Kv1.2 was found in several patients with severe drug-resistant epilepsy. The P405L mutant shows a significant reduction in current density compared with WT (8). The p.255_257del mutation also leads to a loss of channel function. Molecular modeling indicated a repositioning of critical arginine residues in the voltage-sensing domain of the channel, which affects the normal channel function (18). These loss-of-function mutations lead to hyperexcitable neuronal membranes and repetitive neuronal firing due to impaired repolarization (25).

KCNA2 gain-of-function variants are observed in patients with more severe phenotypes such as developmental problems, ataxia and cerebellar atrophy (17). Several sites of gain-of-function mutation have been identified. Pantazis et al. found that F302L mutation may affect one or both aspects of Kv1.2 regulation, potentially exerting differential effects on cellular excitability. Kv1.2 channels with F302L exhibit enhanced inactivation, open faster and require less membrane depolarization to open as a gain-of-function effect. The enhanced KV1.2 inactivation seems to be dominant *in vivo* (26). The probability of the E236K variant increases the likelihood of Kv1.2 channel opening in cortical fast-spiking inhibitory interneurons, promoting membrane hyperpolarization and inhibiting neuronal discharge, reducing GABA release and network disinhibition. Kv1.2 gain-of-function can increase Nav channels’ availability to maintain high excitatory neuron firing rates (17). Gain-of-function mutations of Kv1.2 are sensitive to the Slc7a5-mediated silencing, and when co-expressed with Slc7a5, these mutations may manifest as loss-of-function effects (16). These findings suggest that the symptoms of channel gain-of-function may be modulated by external factors, resulting in varying outcomes.

Treatment methods for epilepsy include gene therapy and drug interventions that target specific types of neurons. In some patients who have drug-resistant epilepsy, a high-fat, low-carbohydrate ketogenic diet is the only treatment effective to reduce seizure frequency (14). Genetic therapy that regulates neuronal excitability in a regional and cell type-specific manner may target these pathologies, particularly in overactive neurons where early gene promoters alter the expression of the Kv1.1 protein. The combination of cfos and EKC (an engineered Kv1.1 potassium channel, with an Ile400Val mutation to bypass posttranscriptional editing and facilitate recovery from inactivation, as described Qiu et al.) has been proven to be highly effective in downregulating neuronal excitability and inhibiting spontaneous epileptic seizures following chemo convulsants (24). 4-Aminopyridine has recently emerged as a promising treatment for encephalopathy patients with gain-of-function KCNA2 mutations (23). 4-AP prolongs action potential duration and increases neurotransmitter release, thus silencing hyperexcitability and improving epilepsy outcome. 4-AP has been shown to enhance cerebellar output and reduce ataxia in some patients with episodic ataxia type 2, possibly by restoring Purkinje cells firing (17).

## KCNB1

KCNB1 encodes the pore-forming and voltage-sensing *α*-subunit of the voltage-gated potassium (K+) channel subfamily 2 (Kv2.1) (9). Kv2.1 is expressed in both excitatory and inhibitory neurons throughout the brain and contributes to the delayed-rectifier potassium currents, mostly in hippocampal and cortical pyramidal neurons (5). Additionally, Kv2.1 plays an essential role in regulating neuronal excitability by participating in action potential repolarization and the dynamic modulation of neuronal activity (27).

*De novo* heterozygous mutations of KCNB1 result in a variety of functional defects, such as loss of ion selectivity, reduced conductance, and dominant adverse effects (28), clinically leading to neurodevelopmental disorders, autism spectrum disorder, and epilepsy of variable severity as reported (27). Marini et al. studied the electroclinical features and outcomes of six patients with *de novo* heterozygous KCNB1 mutations, suggesting that such mutations are not rare (27). Bar and colleagues investigated 36 patients suffering from DEE with seizures or DE with epilepsy and confirmed the connection between mutations of KCNB1 and phenotypic spectrum (29). Truncated KCNB1 variants with haploid sufficiency molecular phenotype cause milder phenotypes (30). These individuals experience less frequent and milder seizures compared to those with missense variants.

The first partial loss of function KCNB1, variant p.Ile199Phe, was identified by Calhoun et al. This mutation was believed to result in epilepsy with centrotemporal spikes, whose symptoms are milder compared to previously reported cases, suggesting a correlation between the degree of KCNB1 protein dysfunction and disease severity (28). In homozygous Kv2.1 knockout mouse models, a reduced seizure threshold was observed (9), while the R312H knock-in mice exhibited a similar phenotype to the null mice (31). These outcomes strengthen the correlation between KCNB1 mutations and the loss of Kv2.1 protein function. Many other reports show that Kv2.1 protein loss-of-function is a typical result of KCNB1 mutations (29). Functional studies have shown that the variant Kv2.1 protein leads to reduced ion selectivity and increased depolarizing inward cation conductance (32). Saitsu and colleagues reported two mutations in the voltage sensor and channel pore domains which cause epileptic phenotypes, also inhibited repeating neuron firing by preventing the production of deep inter-spike voltage (5). However, the precise mechanism remains unclear. In addition to altering excitability, defective channels may cause epilepsy by disrupting neuronal development, potentially leading to abnormal synaptic connectivity (33).

Epilepsy caused by KCNB1 mutations is often highly drug-resistant. Bortolami discovered that Angiotensin II, a key component of IKC (Integrin K^+^ channel Complexes) signaling machinery, could correct morphological defects of primary neurons and enhance their synaptic connectivity in mice with the gene variant KCNB1^R312H^. This variant is commonly observed in children with developmental and epileptic encephalopathies (DEEs) (31). Hu et al. (34) found that valproic acid has a good effect on epilepsy associated with KCNB1 gene mutation. VPA is reported to inhibit excessive endoplasmic reticulum stress and exert neuroprotective effects in acute seizures (35). However, further data from patients with loss or gain-of-function variants are needed to support clinical predictions and treatments strategies.

## KCNC1

KCNC1 encodes the Kv3.1 subunit, which is a typical delayed rectifier channel. Kv3.1 is widely expressed in the neocortex and hippocampus, especially in fast-spiking GABAergic interneurons (36). This channel facilitates rapid voltage-dependent activation and membrane repolarization, enabling high-frequency firing (37).

Mutations in KCNC1 are linked to various neurological disorders, including epilepsy, developmental delay, and ataxia (38). Cameron et al. reported the first cases of KCNC1-associated neuronal disorders, in which the *de novo* variant p.Ala421Val significantly reduces whole-cell current, reflecting a loss of channel function and contributing to the etiology of developmental and epileptic encephalopathy (36). Li et al. hypothesize that Kv3.1 subunits with slight steric hindrance can form functionally impaired tetramers with normal subunits, while more severe steric hindrance may affect the propensity of mutant subunits to compose potassium channel tetramers with the wildtype subunits. Both scenarios lead to loss-of-function of the channel protein, resulting in severe epilepsy and ataxia (38).

Kv3.1 channel gain of function cases have also been reported, with affected K^+^ channels revealed a hyperpolarized shift in voltage-dependent activation and slowed deactivation. In neurons affected by variants, the gain-of-function mutations tend to exert milder effects on excitability compared to the more severe clinical impact of loss-of-function mutations, cases of nonspecific developmental delay and gross motor impairment without epilepsy have been reported (39). *In vitro* analysis has shown that the hyperpolarizing shift is enhanced at febrile temperatures. This result may explain the transient clinical improvement during fever (40). The milder effect of gain-of-function mutation is quite rare in other potassium ion channels.

As genetic testing advances, more *de novo* KCNC1 mutations are being detected. In addition to careful phenotyping, myoclonus epilepsy, and ataxia due to potassium channel mutation (MEAK), patients’ family investigations showed nonignorability of parental chimerism; tests on family members should be added to a clinical gene analysis (41). *In vitro*, few models are available for related research on KCNC2 mutations, though Nengqing et al. successfully established induced pluripotent stem cells (iPSC) from the peripheral blood of MEAK patients with KCNC1 mutations, which proved to be a powerful tool for study (13). However, targeted therapy directed at KCNC1 remains undiscovered. The small molecule Kv3 activator RE01 partially rescued electrophysiological defects, suggesting that pharmacological activation of Kv3.1 activity may offer a treatment option for KCNC1-associated epilepsy patients (42).

## KCND2

KCND2 encodes voltage-gated potassium channel Kv4.2, a major pore-forming subunit involved in the somatodendritic subthreshold A-type potassium current channel (43). Kv4.2 channels have dominant positions in hippocampal CA1 pyramidal cells and cerebellum granule cell layer of anterior lobules (44).

A reduction in the number of channels on the cell membrane leads to a loss of neural function. Peter et al. concluded that Kv4.2 potassium channels were decreased in heterotopic neurons, leading to increased excitability and reduced seizure thresholds, particularly in cases associated with brain malformations (10). The importance of Kv4.2 in regulating neuronal network excitability and dendritic spine morphology has been confirmed by mouse models. Kv4.2 heterozygous mice exhibited elevated theta power and increased spike frequency under basal conditions (45), seizure thresholds decrease correlated with brain malformations in methylazoxymethanol-exposed rats which lack functional A-type Kv4.2 channels (10). Lee et al. reported a gain-of-function Kv4.2 variant in twin patients with both autism and epilepsy. They discovered that p.Val404Met mutation in Kv4.2 constructed slowed inactivation in both homozygous and heterozygous, leading to impaired closed-state inactivation in the presence of KChIP3a (43). Furthermore, microRNA miR-324-5p, which is downregulated during seizures, decreases Kv4.2 expression and contributes to seizure onset. Antagonizing miR-324-5p can protect neurons and suppress seizures (46).

While Kv4.2 loss-of-function mutations are reported to cause refractory temporal lobe epilepsy (47), the gain of function of Kv4.2 has been proven to result in epilepsy. Lin et al. described that the V404M mutation in KCND2 enhanced the inactivation of closed channels, leading to more significant resting inactivation and higher basal excitability. The clinical phenotype in patients with the V404M is more severe than the mild phenotype of Kv4.2 gene knockout mice, indicating that it is more difficult to compensate for gain-of-function mutations that alter essential gating characteristics such as closed-state inactivation.

Lin et al. described the V404M mutation in KCND2 enhances inactivation of closed channels, leading to more significant resting inactivation and higher basal excitability. Patients with V404M have a more severe clinical phenotype than the mild phenotype of Kv4.2 gene knockout mice, suggesting that gain-of-function mutations that alter essential gating properties like closed-state inactivation are more difficult to compensate for (44). The voltage dependence of activation V404L and V404M is similar to Kv4.2 WT; current amplitudes increase when co-expressed with KChIP2 or DPP6 (47).

In addition to miRNA antagonists mentioned above, miRNA antagonists which are mentioned above, studies have found that saikosaponin A may be a targeted treatment of KCND2-related epilepsy by upregulating Kchip1 and Kv4.2 expression, significantly reducing the frequency and duration of spontaneous recurrent seizures. However, direct evidence confirming the role of Kv4.2-mediated transient inactivating K^+^ currents (IA) in this process is still lacking (48).

## KCNQ2 and KCNQ3

KCNQ2 and KCNQ3 genes encode Kv7.2 and Kv7.3 heterotetramers, which contribute to the molecular heterogeneity of the M current (49), a non-inactivating voltage-dependent potassium current that plays a key role in regulating neuronal excitability and limiting repetitive neuronal firing (50). Kv7.2 protein subunits can connect with Kv7.3 to form heterotetrametric channels (51). Kv7.2 and Kv7.3 channels are expressed widely at the plasma membrane of the axon initial segment (AIS) and distal axons (52). Mutate Kv7.2 or Kv7.3 channels mainly cause self-limited or benign epilepsy in neonates and also lead to early onset epileptic encephalopathy (52).

Pathogenic loss-of-function mutations in KCNQ2 have been associated with epilepsy since 1998 (53). Dysfunction of Kv7.2 channels is proven to lead to benign familial neonatal epilepsy (BFNE). It was proposed that a mere 25–50% decrease in the M-current was enough to cause pathology (50). Mutate Kv7.2 channels are also widely found in patients with neonatal epileptic encephalopathy (54). KCNQ2 ablation or loss-of-function at subthreshold membrane potentials leads to increased neuronal excitability in neocortical layer 2/3 pyramidal neurons, unexpectedly resulting in a larger action potential amplitude (55). Chokvithaya et al. identified two novel variants, N258K and G279D, located in the pore loop domain of Kv7.2. Both mutations were confirmed to cause loss-of-function and dominant-negative effects in patch-clamp experiments. The G279D variant at the Gly279 position, a signature sequence of the selectivity K+ filter, leads to a loss of channel function. Channels with the N258K variant may change the membrane potential to return to the resting state much more slowly because of increased membrane capacity in swollen cells (51). The loss of the KCNQ2/3 channel function may result in unlimited Nav1.6 activity. Thus, Carbamazepine, which reduces sodium channel activity, has been proposed as a treatment for KCNQ2 encephalopathy (55).

The M240R variant is the first reported missense loss-of-function pathogenic variant in Kv7.3 channels associated with benign familial neonatal epilepsy (BFNE). This mutation mainly affects voltage sensitivity, other variants that reduce current density have also been reported (56). Maghera et al. revealed that the Kv7.3 T313I variant when assembled with Kv7.2, suppresses currents but has minimal impact on gating parameters (57). KCNQ3 variants appear to be more tolerable than KCNQ2 because no individuals carrying KCNQ2 variants in homozygosity have been reported. In contrast, KCNQ3 variants have been identified in patients with developmental delay and neonatal seizures, suggesting that KCNQ3 variants are compatible with life. On a biochemical level, KCNQ2 deficiency results in a substantial reduction in KCNQ3 and KCNQ5 protein levels, while loss of KCNQ3 only leads to a modest reduction of other KCNQ channels (58).

Both loss-of-function and gain-of-function mechanisms in Kv7.2/Kv7.3 currents can lead to epileptic phenotypes in humans, these phenotypes likely result from dynamics of early activity patterns required for normal brain development (59), the differences in intrinsic biophysical properties or regulatory partners at each subcellular site may also contribute to this phenomenon (60). Devaux et al. identified that p.V175L was a gain-of-function Kv7.2 mutation, and patients with p.V175L mutation appeared to have early onset epileptic encephalopathy, with similar epileptic features to those patients with loss-of-function mutations. The p.V175L mutation increased the surface expression of homomeric mutant channels. This effect may contribute to the increase in current density observed in homogeneous mutation channels, which likely contributes to the observed increase in current density (59). Miceli et al. revealed four mutations, R144Q, R201C, and R201H in Kv7.2 or R230C in Kv7.3 stabilized the activated state of the channel, producing gain-of-function effects. These mutations resulted in neuronal hyperexcitability and severe epileptic phenotypes. They concluded that changes in network interactions, rather than intrinsic cell properties, may be the primary contributor to the observed hyperexcitability in neurons (60).

Loss of function in M-current channels leads to neuronal hyperexcitability (61), identification and development of novel potent. Selective channel openers based on pharmacological activation of Kv7 may lead to a practical therapeutic approach with an improved safety window for antiepileptic and anti-nociceptive effects. Effective KCNQ openers like flupirtine, the structural derivatives of retigabine, were withdrawn one after another due to side effects (61). More drugs are under development. Cannabidiol found in the *Cannabis sativa* plant shifts the voltage dependence of Kv7.2/7.3 channels in the hyperpolarizing direction thereby enhancing the current (62). SCR2682 is reported to selectively and potently activate Kv7 channels and reverse epileptic seizures in rodents (63). Importantly, channel openers may worsen symptoms in patients with gain-of-function Kv7 mutations. Both loss and gain of function variants in Kv7.2/Kv7.3 channels lead to epilepsy, even with similar symptoms (59). The improper use of channel openers could result in heightened neuronal excitability and more severe epileptic manifestations. Thus, it is critical to assess the underlying channel function before initiating therapy.

## KCNH1 and KCNH5

KCNH1 gene encodes Kv10.1 voltage-gated potassium channel, a member of the subfamily H of potassium channels, and KCNH5 encodes Kv10.2, belonging to the ether-a`-go-go (EAG) family. Kv10.1 and Kv10.2 are highly expressed in the human brain and essential for brain development (64). These channels notably contribute to membrane potential regulation and action potential repolarization (65). EAG1 and EAG2 contain a Per-Arnt-Sim (PAS) domain in their N-terminus and a cyclic nucleotide-binding domain (CNBD) in the C-terminus. Many pathogenic mutations localize to these unique domains (66). As reported, dysfunction of the EAG protein resulting from KCNH mutations leads to epileptic encephalopathies and ASD (67).

Epilepsy is a prominent phenotypic feature in most individuals with KCNH1-related syndromes (68). Tian et al. found two novel epilepsy-related pathogenic missense variants of KCNH1 in three individuals. They pointed out that patients who have multiple manifestations of seizure types, severity, and response to treatments with a low level of mosaic/somatic variant or a weaker effect on KCNH1 function of the inherited variant may lead to isolated epilepsy phenotype (64). The molecular sub-regional location of variants is also critical. Ma et al. found that p.Val713Glu mutation of KCNH1 leads to a gain of protein function, probably by inducing a conformational change at the C-terminus or interrupting Ca calmodulin binding, which inhibits channel activity as its location shows, that potassium conductance on the membrane increases due to the mutation and finally results in clinical epilepsy phenotype, such as infantile-onset epilepsy. Treatments targeted to KCNH1-related epilepsy remain rare. Two kinds of C-terminally amidated peptides containing an inhibitor cystine knot motif have been identified as hEAG1 inhibitors and presented a novel mode of action by targeting both the activation and inactivation gating of the gain-of-function channels (69). Benzodiazepine phenytoin, phenobarbital, and valproate have reports of being effective for epilepsy (64).

The genotype–phenotype correlation in KCNH5-related epilepsy has been reported several times. Both gain and loss-of-function mutations in the Kv10.2 channel may lead to epilepsy, probably by altering the excitability of different neuronal types. Happ et al. described 9 individuals with p.Arg327His variant located in the voltage-sensing domain of Kv10.2. The variant leads to a gain of channel function, thus resulting in developmental and epileptic encephalopathy. They also hypothesize that other KCNH5 voltage-sensing and pore domain variants lead to epilepsy through a similar gain-of-function mechanism (70). In excitatory neurons, gain-of-function mutations enhance afterhyperpolarization of action potentials, leading to an increased firing frequency that increased potassium channel activity within negative voltage domains may also lead to additional hyperpolarization and cation current activation, resulting to secondary depolarization. In contrast, when gain-of-function mutations affect inhibitory neurons, they increase basal K^+^ conductance and reduce input resistance of the cellular membrane, disrupting neuronal network inhibition (71). Galán-Vidal et al. found loss-of-function Kv10.2 mutant channels encoded by N856H slowly activating outward current, which was quite noticeable compared to WT Kv10.2 channels. Loss-of-function mutations alter the resting membrane potential and the repolarization process, thus leading to epilepsy and autism. Few treatments are available for KCNH-related epilepsy. Valproate enhances GABAergic transmission, while terbutaline could improve potassium transport. Both of them control epilepsy well (65). Valproic acid and lamotrigine are also used to treat KCNH-related epilepsy.

## Conclusion

With the continuous progress of gene detection technology, the identification of genes associated with epilepsy is progressing rapidly through techniques such as whole genome sequencing. *De novo* mutations and mosaicism have been implicated as partial explanations for the mechanisms underlying some epilepsy cases, though many mechanisms remain unexplored. The induced pluripotent stem cell (iPSC) technology provides a reliable *in vitro* model, and the gene knockout mouse models effectively simulate ion channel dysfunction. As understanding of the pathogenesis of epilepsy deepens, novel treatment strategies are being developed. Ion channel openers have been employed in clinical settings, and gene-targeted therapies are increasingly being introduced.

There are still areas requiring further investigation. Currently, most *in vitro* experiments are conducted at room temperature, so further simulations of the human body are needed. In addition, most mouse models used in experiments are male. We all know that due to the influence of hormones, the pathogenesis of epilepsy in males and females is slightly different, so the gender of the experimental mice should also be considered. Channel gain of function may not lead to neural function increasing, and channel variations with epileptic phenotypes may not be the leading cause of symptoms in some cases, as altered synaptic density, KCHIPs, or other dysfunctional neurological components can also cause increased neural excitability. As technology advances, further *in vitro* and animal studies will help confirm the role of mutated genes in epilepsy. We believe that precise diagnosis and treatment of epilepsy-related to voltage-gated potassium channels will be achieved 1 day.
